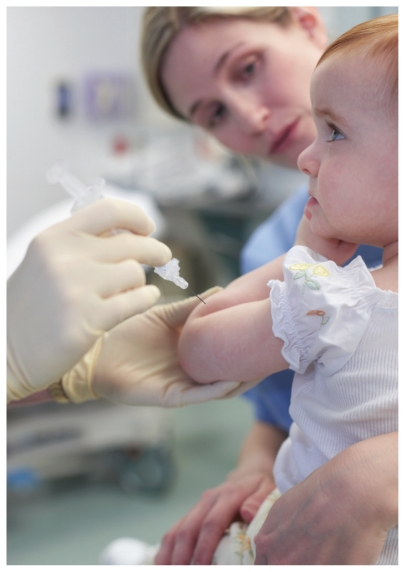# Diminished Protection?: Early Childhood PCB Exposure and Reduced Immune Response to Vaccinations

**DOI:** 10.1289/ehp.118-a445a

**Published:** 2010-10

**Authors:** Julia R. Barrett

**Affiliations:** **Julia R. Barrett**, MS, ELS, a Madison, WI–based science writer and editor, has written for *EHP* since 1996. She is a member of the National Association of Science Writers and the Board of Editors in the Life Sciences

Polychlorinated biphenyls (PCBs) constitute a class of persistent organic pollutants suspected or known to cause adverse health effects. Among these effects are immune system dysfunctions that may hinge on both the magnitude and the timing of PCB exposure. A new study uses the backdrop of routine childhood immunizations to explore the developmental immunotoxicity of PCBs and finds that higher PCB exposure in toddlerhood is associated with reduced antibodies against diphtheria and tetanus later in childhood **[*EHP*** **118(10):1434–1438; Heilmann et al.]**.

The study took place in the Faroe Islands, midway between the Shetland Islands and Iceland in the North Atlantic, and involved 587 children from a 1999–2001 birth cohort. A traditional diet including pilot whale blubber, consumed by some but not all Faroese, creates a wide range of PCB exposures for this population. To assess the transfer of PCB from a mother to her child, maternal blood samples were taken in week 32 of pregnancy. The mothers also provided milk samples at 4–5 days after birth.

Based on the routine vaccination schedule, children were immunized against diphtheria and tetanus at 3, 5, and 12 months, with a booster at 5 years. Approximately one-fifth of the children had blood drawn at 18 months, and blood samples were drawn before the booster shots at age 5 years for 532 children and 7 years for 464 children. PCB concentrations were assessed in blood and milk samples, and diphtheria and tetanus antibodies were measured in children’s blood at ages 5 and 7.

Analysis revealed inverse relationships between PCB concentrations at different time points and antibody concentrations. The associations between concomitant measurements were not significant at either 5 or 7 years. However, higher PCB concentrations in mother’s milk samples collected after birth and in children’s blood samples at 18 months were clearly associated with lower levels of diphtheria antibodies in the children at age 5. When PCB concentrations at 18 months were estimated for the entire cohort based on known levels at birth and at 5 years paired with breastfeeding duration and PCB concentrations measured in blood samples from a subset of children at 18 months, this relationship became even stronger for diphtheria at both 5 and 7 years, and a similar relationship for tetanus antibody concentration at age 7 became significant.

The authors point out that early-life PCB exposure may increase the risk of incomplete protection against diphtheria and possibly tetanus even if a child receives a full schedule of vaccinations. But the implications of the results extend beyond vaccination because diphtheria and tetanus immune response reflects the efficacy of the immune system against a broad array of infections.

## Figures and Tables

**Figure:**